# Post-Conflict Affiliation by Chimpanzees with Aggressors: Other-Oriented versus Selfish Political Strategy

**DOI:** 10.1371/journal.pone.0022173

**Published:** 2011-07-20

**Authors:** Teresa Romero, Miguel A. Castellanos, Frans B. M. de Waal

**Affiliations:** 1 Department of Cognitive and Behavioral Science, The University of Tokyo, Tokyo, Japan; 2 Living Links, Yerkes National Primate Research Center, Emory University, Atlanta, Georgia, United States of America; 3 Department of Methodology of Social Sciences, Faculty of Psychology, Complutense University of Madrid, Madrid, Spain; Texas A&M University, United States of America

## Abstract

Consolation, i.e., post-conflict affiliation directed from bystanders to recent victims of aggression, has recently acquired an important role in the debate about empathy in great apes. Although similar contacts have been also described for aggressors, i.e., appeasement, they have received far less attention and their function and underlying mechanisms remain largely unknown. An exceptionally large database of spontaneous conflict and post-conflict interactions in two outdoor-housed groups of chimpanzees lends support to the notion that affiliation toward aggressors reduces the latter's aggressive tendencies in that further aggression was less frequent after the occurrence of the affiliation. However, bystander affiliation toward aggressors occurred disproportionally between individuals that were socially close (i.e., affiliation partners) which suggest that it did not function to protect the actor itself against redirected aggression. Contrary to consolation behavior, it was provided most often by adult males and directed toward high ranking males, whereas females engaged less often in this behavior both as actors and recipients, suggesting that affiliation with aggressors is unlikely to be a reaction to the distress of others. We propose that bystander affiliation toward aggressors may function to strengthen bonds between valuable partners, probably as part of political strategies. Our findings also suggest that this post-conflict behavior may act as an alternative to reconciliation, i.e., post-conflict affiliation between opponents, in that it is more common when opponents fail to reconcile.

## Introduction

Despite the advantages of group living, individuals in many animal societies have only partially overlapping interests. Conflicts of interest between group members occur in many contexts, such as competition for limited resources [1], [2] and disagreement about decisions [3], which might be expressed through dyadic or higher-order contests, putting benefits at risk. The resolution of aggressive conflict is therefore of great survival value. Various conflict resolution strategies have been reported for over thirty primate species, both in captivity and in the wild [4]–[6], as well as for several non-primates [7]–[11]. Of particular interest are “triadic” conflict resolution strategies (i.e. initiated by individuals not directly involved in the conflict) since they may require knowledge of the social relationship among other group members [12]. Their study may provide valuable information about the evolution of cognitive and emotional mechanisms in primate and other animals [13].

The triadic post-conflict behavior labeled consolation, i.e. affiliative contact initiated by uninvolved individuals to recent victims of aggression, has recently acquired an important role in de debate about animal empathy since its limited distribution has been interpreted as a reflection of the empathy level required to reassure distressed parties [13]–[15]. Although similar contacts are also offered by bystanders to aggressors during the post-conflict period (i.e. appeasement, [16]) they have received far less attention and their functional significance and cognitive and emotional implications are largely unknown. In fact most, if not all, of the suggested functions for appeasement are derived from the theoretical framework developed for contacts directed to victims [17]. Although both aggressor and victim are affected by the negative consequences of aggression, victims are likely to experience higher levels of anxiety [18], [19] and thus may have a greater need of reassurance. On the bystander's side, approaching and contacting the aggressor may be more meaningful with respect to certain functions than contacting the victim (i.e. to provide encouragement; [17]), although it may also entail a higher risk of receiving aggression [20]. Therefore, bystander affiliation toward aggressors and victims may be qualitatively different and should therefore be investigated separately.

A critical aspect to understand the underlying mechanisms of appeasement is to understand its effect on others. Appeasement is a functional term which carries the implicit assumption of reducing aggressive tendencies in a potential aggressor. According to the self-protection hypothesis, post-conflict affiliation toward aggressors provides direct and immediate benefits to bystanders, hence should be performed most often by likely targets of aggression [21]. A similar decrease on post-conflict aggressive tendencies would be expected, however, if affiliation toward aggressors were part of the policing strategies documented for some primate species [22]. These strategies are typically performed by a small subset of individuals, i.e. powerful high ranking individuals [23], [24], and they aim to reduce the spread of the aggression and/or social tension within the group.

An alternative functional hypothesis proposes that bystander affiliation toward aggressors serves to repair the relationship between both former opponents, which was disrupted by the previous conflict. It has been demonstrated that when former opponents reunite soon after the end of an aggressive conflict (c.f. reconciliation,[25]) their mutual tolerance is restored to baseline levels [5], [6]. However, approaching a former opponent may be risky because aggression may resume [26]. In such cases, bystanders may function as mediators, reconciling with the aggressor on behalf of the victim [27]–[30]. According to this hypothesis affiliation should be provided mostly by the former victim's friends or kin, and it would require so-called *triadic awareness*, or knowledge of third-party relationships [24], [31], [32].

Finally, bystander affiliation toward aggressors has been proposed to function as a mechanism to alleviate the aggressors' stress caused by the previous conflict, similar to contacts directed to victims [14]. In this case, the bystander's motivation for offering affiliation is considered to be empathy, and the post-conflict affiliation should involve individuals with whom the opponent shares a close relationship given that empathic responses are greatly facilitated by similarity, familiarity, and social closeness [33], [34].

The quality of the relationship between the individuals involved in the post-conflict interaction has been proven to be a critical factor in determining their occurrence and function [4], [6]. Furthermore, the relationship between the bystander and the opponents may determine the cost and benefits of the triadic post-conflict interactions and hence its examination is critical to understand their function and determinants [17], [35]. The effect of relationship quality on contacts directed to aggressors, however, has received very little attention. To date only one great ape study has investigated the relationship between bystanders and opponents, finding a disproportionate representation of both their close social partners and the opponent's close social partners among bystanders [30]. Even though these findings give partial support to two of the suggested functional hypothesis (i.e. stress-alleviation and relationship-repair hypotheses), it cannot exclude the policing hypothesis given that the study failed to address the social role of bystanders. Furthermore, affiliation toward aggressors may also be affected by several other factors, such as the characteristics of the conflict itself, or the occurrence of alternative conflict resolution strategies, and yet no study has examined simultaneously the impact of these factors in the occurrence of affiliation from bystanders toward aggressors.

Here we used an unusually large database, which sample size is many times larger than that of any previous study on animal conflict resolution strategies, to address all the above questions in a single analysis. A total of 3,003 aggressive conflicts and post-conflict periods were used to investigate the determinants of third-party appeasement among chimpanzees (*Pan troglodytes*), exploring in particular the quality of the relationship between bystanders and conflict opponents. We examined whether aggressors are contacted mostly (1) by individuals that they tend to target aggressively (as predicted by the appeasement hypothesis), (2) by high ranking conflict managers (as predicted by the policing hypothesis), (3) by their opponent's close associates and kin (as predicted by the relationship repair hypothesis), or (4) by close associates and kin (as predicted by the stress-alleviation hypothesis). We also measured individual characteristics of involved individuals, the characteristics of the previous conflict, the relation between conflict participants, as well as the co-occurrence of other post-conflict affiliative interactions, such as reconciliation and consolation. In addition, we test the assumed function of appeasement behavior in chimpanzees (i.e. to reduce aggressive tendencies in potential aggressors), which we investigated by measuring the bystanders' likelihood of receiving further aggression from the individuals they aim appeasement at.

## Results

### Social determinants

The effect of a variety of variables on the likelihood of third-party appeasement (i.e. the first affiliative contact made by a bystander toward the former aggressor) was measured using Generalized Linear Mixed Models (GLMM). When analyzing the variables related to the characteristic of the previous conflict, the only variable remaining in the best model was the co-occurrence of reconciliation between both opponents (ß = 0.420, p<0.001). Reconciliation had a negative effect on appeasement, which means that appeasement was more likely when the previous opponents had failed to reconcile.

### Triadic Relations

**Figure 1:**
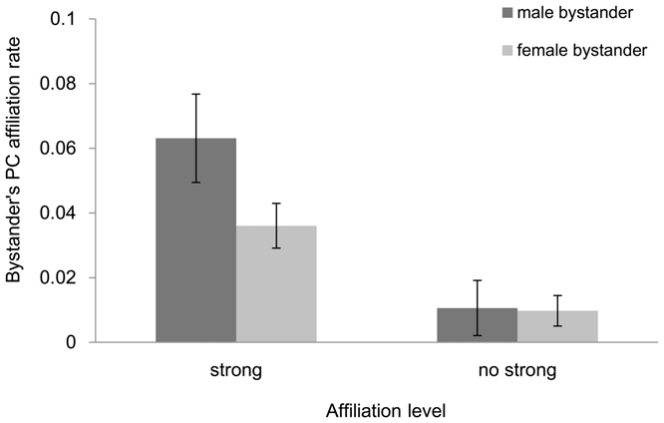
Bystander affiliation rate in relation to bystander's sex and affiliation level between bystanders and aggressors. Post-conflict bystander affiliation rate was calculated as the number of affiliations corrected by the total number of opportunities to receive affiliation. Bars represent mean post-conflict affiliation rates ±95% confidence intervals.

We investigated how relational variables between bystander and aggressor, and bystander and victim determined the occurrence of third-party appeasement by running GLMM. While none of the victim's variables affected the occurrence of appeasement, the affiliative relation between bystander and aggressor and the interaction between affiliation level and bystander's sex remained significant in the best model (Table 1). Appeasement was directed more often at individuals with whom the bystander had a strong affiliative tie (strong vs. no-strong affiliation: ß = 0.578, p<0.001, Table 1). Furthermore, aggressors were more likely to be contacted by male than female close social partners (interaction between affiliation level and bystander's sex, Figure 1, Table 1).

**Table 1:** Variables in the best GLMM explaining the occurrence of appeasement according to bystanders and aggressors characteristics.

variables	*ß*	SE	*z*	*P*	odds ratio	odds ratio IC (95%)
**Fixed**						
Intercept	−4.934	0.388	−12.716	<0.001		
Affiliation level	0.578	0.110	5.235	<0.001	1.78	1.43–2.21
Bystander's sex	1.073	0.441	2.432	0.015	2.92	1.23–6.94
Aggressor's sex	1.575	0.417	3.769	<0.001	4.83	2.12–10.9
Aggressor's rank						
*medium vs. high*	0.752	0.495	1.52	0.128	2.12	0.80–5.59
*low vs. high*	0.112	0.362	0.31	0.756	1.12	0.54–2.27
Affiliation Level×Bystander's sex						
*strong×bystander male*	0.814	0.222	3.665	<0.001	2.25	1.46–3.49
Aggressor's rank×Aggressor's sex						
*aggressor rank medium×male*	−1.386	0.583	−2.375	0.017	0.25	0.08–0.78
*aggressor rank low×male*	−1.923	0.449	−4.283	<0.001	0.14	0.06–0.35
Aggressor's rank×Bystander's sex						
*aggressor rank medium×bystander male*	−0.852	0.364	−2.34	0.019	0.42	0.21–0.87
*aggressor rank low×bystander male*	−0.647	0.305	−2.124	0.033	0.52	0.28–0.95
Bystander's sex×Aggressor's sex						
*bystander male×aggressor male*	−0.856	0.268	−3.187	0.001	0.42	0.25–0.71

### Sex differences

**Figure 2:**
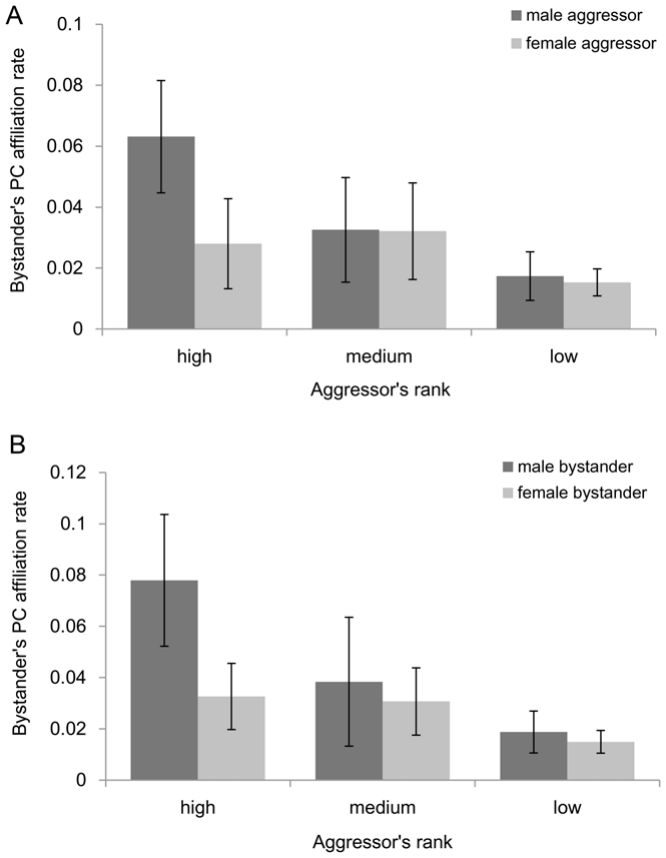
Bystander affiliation rate in relation to the aggressor's rank and (a) aggressor's sex and (b) bystander's sex. Bars represent mean post-conflict affiliation rates ±95% confidence intervals.

Sex of the involved individuals had a significant impact on the likelihood of appeasement. Aggressor's sex, bystander's sex and the interactions between aggressor's sex and aggressor's rank, bystander's sex and aggressor's rank, and aggressor's sex and bystander's sex remained significant in the best model (Table 1). Overall, male aggressors received more appeasement than female aggressors (ß = 1.575, p<0.001), and in particular, high ranking male aggressors were contacted more frequently than other group members (interaction between aggressor's sex and aggressor's rank, Table 1, Figure 2A).

**Figure 3:**
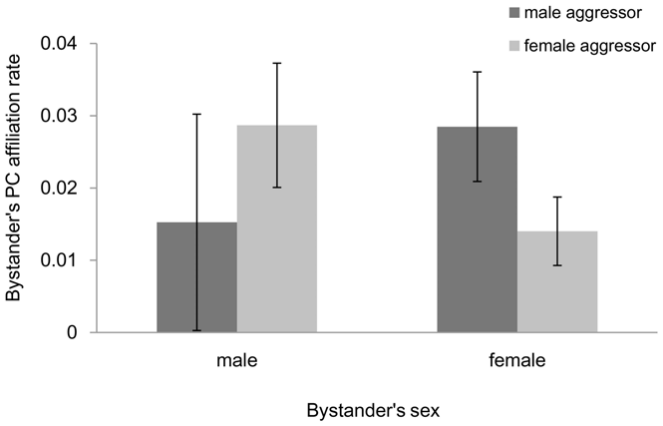
Bystander affiliation rate in relation to the aggressor's sex and bystander's sex. Bars represent mean post-conflict affiliation rates ±95% confidence intervals.

On the bystander's side, male bystanders provided appeasement more often than did females (ß = 1.073, p = 0.015), and they did offer appeasement mostly to high ranking aggressors (interaction between bystander's sex and aggressor's rank, Table 1, Figure 2B). Furthermore, the interaction between bystander sex and aggressor sex significantly improved the model which suggests that while males affiliated with both male and female aggressors, bystander females contacted mostly male aggressors (interaction between bystander's sex and aggressor's sex, Table 1, Figure 3).

### Further aggression

Aggressors directed aggression to uninvolved bystanders in 3.82% of post-conflict periods after the original aggression. The occurrence of further aggression in PC's without third-party appeasement, consolation or reconciliation was compared with the occurrence of further aggression after the occurrence of third-party appeasement by running GLMM analyses. After the occurrence of appeasement, aggressors tended to direct aggression less often than when appeasement did not occur (ß = 1.073, p = 0.015).

## Discussion

The present multivariate study provides evidence that post-conflict bystander affiliation toward aggressors reduces the latter's aggressive tendencies. Our findings also suggest that this post-conflict behavior could act as substitute reconciliation in that it is more common when opponents have failed to reconcile. Bystander affiliation toward aggressors is provided most often by adult males and directed toward high ranking males, whereas females engage less often in this behavior both as actors and recipients.

In both study groups, aggressors were more likely to be contacted by a bystander when they had not reconciled their conflict than when they did. This type of post-conflict interaction therefore may function as an alternative to reconciliation. The interdependency of reconciliation and other post-conflict interactions has been previously documented [36]–[38] and it is expected to occur when their functions overlap [17]. However, to be a true alternative to reconciliation, i.e. repairing relationship between opponents, bystanders are expected to have a close tie with the victim, such as kin or close social partners. Indeed, a recent study on wild chimpanzees has shown that when friends of the victim affiliated with the aggressors after a conflict, the tolerance levels between former opponents were restored to baseline [30]. In the present study, however, the nature of the relationship between the victim and the bystander did not affect the occurrence of contacts directed to aggressors suggesting that the main function of bystander affiliation toward aggressors was not to substitute for reconciliation.

In contrast, the relationship between aggressors and bystanders had a strong impact on the occurrence of bystander affiliation toward aggressors, since aggressors were more likely to received affiliation from their closest associates during the post-conflict period. The relationship between the aggressor and the third party is thought to be important when affiliation serves to alleviate the aggressor's distress. Since aggressors, and not only victims, may experience post-conflict stress due to the uncertain of their future relationship with the victim [19], [39], they may also need reassurance. In this case, bystanders providing post-conflict affiliation are likely to share a valuable relationship with the aggressor since such partners are more likely to be responsive to each other's distress [14], [15], [34], [40]. Aggressor affiliation with close social partners has also been observed in other chimpanzee [30] and bird (*Corvus frugilegus*, [41]) studies. However, the only study that has investigated the effect of bystander affiliation on aggressor's stress levels found no evidence for stress-reduction, which suggests that alleviation of the aggressor's distress is unlikely to be the main function of this post-conflict behavior [20]. Furthermore, since it has been proposed that empathy evolved from the context of maternal care, females are expected to be more sensitive to or more accurate in evaluating signs of distress in others [34], [42]. Indeed, contacts directed to chimpanzee victims of aggression, which have been proven to reassure distressed parties and suggested to rest on empathic arousal in the actor, are mainly provided by females [15]. In contrast, chimpanzee aggressors were more likely to be contacted by their male partners, suggesting that offering affiliation to aggressors is unlikely to be a reaction to distress. Since the stress-alleviation function applies mainly to close social partners, further research should examine the stress alleviation effect of bystander affiliation directed to aggressors according to the nature of the relationship between the bystander and the aggressor.

Consistent with the notion that affiliation from bystanders toward aggressors had an appeasement effect, chimpanzee aggressors redirected aggression less often after being contacted by a third party than when they were not. In social groups, the negative consequences of aggression can spread beyond the two original contestants. Third parties may give agonistic support to either aggressor or victim [43], and former opponents may also redirect aggression to other group members [39], [44]. Post-conflict bystander affiliation toward aggressors has been thought to function as a mechanism to reduce aggressors' aggressive tendencies. Previous research in chimpanzees and gorillas (*Gorilla spp.*) further support this idea, because it has been proven that affiliation with aggressors reduces the likelihood of further aggression either at group [45] or at individual level [21]. In agreement with this notion, aggressors who were more likely to redirect aggression, i.e. high ranking males (percentage of further aggression performed by high ranking males, FS1 = 64%, FS2 = 50.8%) were also more likely to receive affiliative contacts during the post-conflict period.

Even though bystander affiliation had a negative effect on the occurrence of further aggression, it is unlikely that it functions as a direct mechanism of self-protection for bystanders. The self-protection hypothesis would suggest that affiliating with former opponents could provide protection to bystanders by reducing their likelihood to receive redirected aggression. Thus, bystanders should selectively direct affiliation to those conflict participants who more often gave aggression to them. In contrast, our findings show that levels of aggression received by third parties from aggressors did not affect the participation of bystanders in appeasement contacts. During post-conflict periods, frequent targets of aggression were not more likely to offer affiliation to aggressors than non-targets of aggression. Furthermore, aggressors were more likely to be contacted by adult male chimpanzees, which are unlikely targets of redirected aggression.

Bystander affiliation toward aggressors thus might function to prevent the diffusion of conflict throughout the group, in which case it could be considered part of the repertory of policing strategies display by chimpanzees. Previous research on both captive and wild chimpanzee populations have pointed out the special social role of adult dominant male chimpanzees in containing and terminating open conflicts. Adult male chimpanzees often intervene in on-going aggressive conflicts and perform pacifying interventions [24], [46]. It would be expected that such policing strategies were also displayed during the post-conflict period, especially when the risk of further aggression is elevated. Our findings, however, give only partial support to this hypothesis. Both, pacifying interventions and bystander affiliation toward aggressors seem to be effective in reducing the spread of the aggression throughout the group. However, while pacifying conflict interventions in chimpanzees and other primates are performed almost exclusively by high-ranking individuals [46], [47], the distribution of bystander affiliation toward aggressors was not affected by bystander's rank since high-ranking individuals did not affiliated with aggressors more often than lower ranking ones.

The fact that appeasing bystanders share a close social relationship with the aggressor and males are the prime performers leads us to suggest that bystander affiliation toward aggressors might function to show support for valuable partners or to strengthen bonds between allies, similar to the post-conflict bystander affiliation described for corvids [41]. There is increasing evidence that chimpanzees and other animals may achieve substantial direct benefits by forming close social bonds with conspecifics [48]–[50]. Chimpanzee male social status is highly influenced by their relationships with other group members and their ability to form and maintain cooperative alliances [24], [51], [52]. Social tolerance, grooming or direct support during open conflicts have been typically described as tactics used to develop such relationships. Post-conflict affiliative contacts toward aggressor might well be part of these strategic investments. Affiliating with an opponent once the aggression has ceased allows individuals to show support for a valuable partner, without facing the risk of intervening in the agonistic conflict. These contacts also may communicate existing alliances to others. Consistent with this notion, valuable potential partners, i.e. high-ranking individuals, were the most frequent targets of bystander affiliation. Future studies should examine whether affiliation toward aggressors is part of a behavioral exchange between partners, and hence, the bystanders would derive benefits by receiving support or other valuable behavior in the future from the aggressor.

In summary, the fact that bystander affiliation toward aggressors was disproportionally aimed at high ranking males and provided mainly by male, makes it unlikely that this behavior reflects empathetic arousal. The findings of the present study also show that although bystander affiliation toward aggressors reduces aggressors' aggressive tendencies, it is unlikely that it functions as a direct self-protective mechanism for the acting bystander. It seems more likely that approaching a former aggressor during the post-conflict period is part of political strategies, either as a policing mechanism to reduce the spread of aggression or to demonstrate support for the aggressor or existing alliances to others.

## Methods

### Ethics Statement

All research reported in this manuscript was approved by the Institutional Animal Care and Use Committee of Emory University (approval number 083-2008Y) and was conducted in strict accordance with the Weatherall Repot on “The use of non-human primates in research” and the “Guidelines for the treatment of animals in behaivoural research and teaching” by the Animal Behavior Society/Association for the Study of Animal Behaviour.

### Study population

Subjects were two socially housed groups of chimpanzees (FS1 and FS2) at the field station of the Yerkes National Primate Research Center in Lawrenceville, Georgia (USA), which is fully accredited by the American Association for Accreditation of Laboratory Animal Care (IACUC approval number 083-2008Y). Chimpanzees lived in large outdoor areas with access to heated sleeping indoor areas. The demographic composition of groups varied slightly during the study period due to births, deaths and several removals for veterinary reasons and management purposes. A more detailed description of the study subjects can be found in Romero & de Waal [35]. Most of the time, both groups included multiple adult males and at least twice as many females. The analyses of this study have been limited to individuals at least 10 years old (i.e. 8 males and 21 females).

### Data collection

Data presented here refers to the period of time from 1992 to 2000 for FS1 and from 1994 to 2000 for FS2. During that period, 90 min controlled observation sessions [53] were conducted regularly, approximately once a week, in both study groups. A trained research technician, Mike Seres, recorded using an all-occurrence sampling technique any affiliative and sexual interaction (i.e., kiss, embrace, grooming, gentle touch, finger/hand-in-mouth, mounting) and agonistic interaction (which by definition include at least one of the following behavior elements: tug, brusque rush, trample, bite, grunt-bark, shrill-bark, flight, crouch, shrink/flinch, or bared-teeth scream, [54], [55]). Additionally, scan samples of state behaviors (e.g. contact-sitting, grooming, play) were taken at regular intervals (i.e. every 5 minutes through 1993 and every 10 minutes in the years thereafter).

Following de Waal & van Roosmalen [25] an interaction was considered an agonistic conflict if at least one of the agonistic patterns previously listed occurred. For each conflict the identities of the initial aggressor and the initial recipient of aggression were recorded along with the intensity, directionality and outcome of the conflict. The intensity was scored as low if the conflict included a threat, chase and/or brusque rush, as medium if it included hit, punch, push and pull, and as high if it involved trample or bite. Unidirectional conflicts were those in which all aggressive behavior was directed toward the initial recipient of aggression and no counter-aggression occurred. Otherwise, conflicts were classified as bidirectional. The outcome of the conflict was recorded as decided if only one of the parties showed signs of submission (e.g. screaming, teeth-baring, fleeing, or pant-grunt) and as undecided in the remaining cases. During the immediate 10 minutes following aggression (i.e. post-conflict period), all affiliative and agonistic interactions involving the former opponents were recorded, as well as the time of the interaction, the identity of the interaction partners and the identity of the initiator of the interactions.

### Data analysis

A total of 3,003 valid 10 min post-conflict (PC) periods were collected (i.e. 1,676 for FS1 and 1,327 for FS2). For the purpose of this study, reconciliation was operationally defined as the first affiliative contact between former opponents after a conflict, appeasement as the first affiliative contact directed from a third party to the initial aggressor, and consolation as the first affiliative contact directed from a bystander to the recipient of aggression. Bystanders were defined as those individuals who were neither involved in the conflict or in any agonistic interaction in a time window of ±2 min from the occurrence of the conflict.

Generalized Linear Mixed Models (GLMM) with a binomial error structure and logit link function were used to examined whether the occurrence of appeasement (i.e. behavior present or absent) was affected by several factors. Conflict characteristics (i.e.; intensity, directionality and outcome), relationship characteristics between aggressors and recipients (i.e.; dominance, kinship, affiliation level), and the occurrence of reconciliation and consolation were entered as fixed variables (Table 2). Dominance was defined by the direction of submissive signals, such as pant-grunt and bobbing movements, and by non-agonistic approach/retreat interactions. Kinship relationships were restricted to mother - infants, maternal siblings, and grandmother - grandchildren. The one adoptive relationship was also treated as kin. A combined measure of four state behaviors collected during scans (i.e. contact sitting, sitting within arm's reach, grooming and mutual grooming) was used to calculate the affiliation level between dyads. The quartile points of dyadic scores for each focal individual were calculated and only dyads with scores higher than the top quartile were considered to have a strong affiliative relationship. Similarly, dyads were classified according to their aggression level. A dyad (between individuals A and B) was named “target of A” if the rate of aggression directed by A against B was in the top quartile of A's aggressive scores. Otherwise, the dyad was labeled “non-target”. The identity of aggressors and recipients of aggression, as well as the study group name (i.e. FS1, FS2) were entered as random variables.

**Table 2:** Variables used in GLMM analyses for the determinants of appeasement.

name	type
Dependent variable	
- Appeasement behavior	Dichotomous (1 = yes, 0 = no)
- Frequency of appeasement	Continuous
**Fixed explanatory variables**	
- Conflict characteristics	
- Outcome	Dichotomous (1 = decided, 0 = undecided)
- Intensity	Ordinal (1 = low, 2 = medium, 3 = high)
- Directionality	Dichotomous (1 = unidirectional, 0 = bidirectional)
- Reconciliation	Dichotomous (1 = yes, 0 = no)
- Consolation	Dichotomous (1 = yes, 0 = no)
- Individual characteristics	
- Sex	Dichotomous (1 = male, 2 = female)
- Rank	Ordinal (1 = high, 2 = medium, 3 = low)
- Relationship characteristics	
- Kinship	Dichotomous (1 = kin, 0 = no kin)
- Affiliation level	Dichotomous (1 = strong, 0 = no-strong)
- Aggression level	Dichotomous (1 = target, 0 = no-target)
**Random variables**	
- Aggressor's, recipient's & bystander's identity	Nominal
- Group	Nominal

To examine the effect of individual characteristics of participants and relationship characteristics between opponents and bystanders on the occurrence of appeasement two different GLMM analyses were performed with the frequency of giving appeasement as a dependent variable. In the first analysis, the frequency of giving appeasement equaled the number of times each potential bystander initiated the affiliative interaction toward a particular aggressor. To correct for the opportunity each potential bystander had to contact the aggressor, we included as an offset variable the number of PCs in which one individual was the aggressor of a conflict, excluding those in which the partner was an involved individual (i.e. the victim or a supporter of either opponent). Individual characteristics of aggressors and bystanders (i.e.; sex and rank) and relationship characteristics between aggressors and bystanders (i.e.; kinship, affiliation level) were input as fixed terms (Table 2). In the second analysis, the frequency of appeasement equaled the number of times each potential bystander offered appeasement when a particular individual was the victim. We corrected for the opportunity to offer appeasement including as an offset variable the number of PCs in which one individual was the victim excluding those in which the partner was an involved individual in the conflict (i.e. the aggressor or a supporter of either opponent). The GLMM was then run including the victims' variables (Table 2). As random terms we included the identity of opponents and bystanders and the group name.

To investigate how the occurrence of further aggression was affected by the occurrence of appeasement a GLMM analysis was conducted. The dependent variable was a binary term (binomial error structure) of whether or not the aggressor attacked the bystander after the occurrence of the affiliation. For PCs in which no affiliation occurred between bystanders and opponents or between opponents the time window expanded to the whole 10 min PC period. PCs in which appeasement co-occurred with other post-conflict affiliative interactions (i.e. reconciliation or consolation) were excluded. The occurrence of appeasement was included as a fixed term and the identity of opponents and bystanders as random terms. For all GLMM analyses, we used a step-up strategy (i.e., fixed factors were added to the model sequentially), and selected the best model based on Akaike's information criterion (AIC). GLMM analyses were run on R version 2.8.1 [56] using the lmer function included in the lme4 package.
